# Are We Ready to Treat Diffuse Large B-cell and High-Grade Lymphoma According to Major Genetic Subtypes?

**DOI:** 10.1097/HS9.0000000000000284

**Published:** 2019-07-31

**Authors:** Annalisa Chiappella, Jennifer Crombie, Anna Guidetti, Umberto Vitolo, Philippe Armand, Paolo Corradini

**Affiliations:** 1 Hematology, Azienda Ospedaliero Universitaria Città della Salute e della Scienza di Torino, Italy; 2 Department of Medical Oncology, Dana-Farber Cancer Institute, Boston, Massachusetts, USA; 3 Division of Hematology, Fondazione IRCCS Istituto Nazionale dei Tumori, Milan, Italy; 4 University of Milan, Italy; 5 Dana-Farber Cancer Institute, 450 Brookline Avenue, Boston, MA, USA.

## Abstract

Diffuse Large B-Cell Lymphoma (DLBCL) is a clinically and biologically heterogeneous disease. The revised Classification of Lymphoproliferative diseases published in 2016 (WHO, 2016) refined the previous DLBLC subtypes and identified four categories: DLBCL not otherwise specified (NOS), other lymphomas of large B cells, high grade B-cell lymphoma, and B-cell lymphoma unclassifiable. High grade B-cell lymphomas include the entities carrying MYC, BCL2 and/or BCL6 translocations or cases with blastoid morphology without DH translocations. This classification also acknowledges the cell of origin (COO) classification, that has only a limited impact on the choice of frontline treatment for DLBCL, as most patients still receive R-CHOP chemoimmunotherapy. Attempts to improve the outcomes of specific subgroups, especially COO groups, have so far had limited success. Newer analyses have further subdivided DLBCL into genomically distinct subsets, not yet incorporated in the WHO classification, which may facilitate targeted approaches to therapy. In this review, we discuss the subgroups that are recognized by the WHO 2016 classification, review the newer genomic data, and speculate on how this could alter the treatment landscape of DLBCL in the future. We also discuss novel approaches to salvage therapy in the broad context of the heterogeneity of DLBCL.

## Introduction

Diffuse Large B-Cell Lymphoma (DLBCL) is a clinically and biologically heterogeneous disease. The revised Classification of Lymphoproliferative diseases published in 2016 (WHO, 2016)^1^ refined the previous DLBLC subtypes and identified four categories: DLBCL not otherwise specified (NOS), other lymphomas of large B cells, high grade B-cell lymphoma, and B-cell lymphoma unclassifiable. High grade B-cell lymphomas include the entities carrying MYC, BCL2 and/or BCL6 translocations or cases with blastoid morphology without DH translocations (Table 1). This classification also acknowledges the cell of origin (COO) classification, further discussed later in this review. At present, this classification has only a limited impact on the choice of frontline treatment for DLBCL, as most patients still receive R-CHOP chemoimmunotherapy. Attempts to improve the outcomes of specific subgroups, especially COO groups, have so far had limited success. However, newer analyses have further subdivided DLBCL into genomically distinct subsets, not yet incorporated in the WHO classification, which may facilitate targeted approaches to therapy. In this review, we discuss the subgroups that are recognized by the WHO 2016 classification, review the newer genomic data, and speculate on how this could alter the treatment landscape of DLBCL in the future. We also discuss novel approaches to salvage therapy in the broad context of the heterogeneity of DLBCL.

**Table 1:**
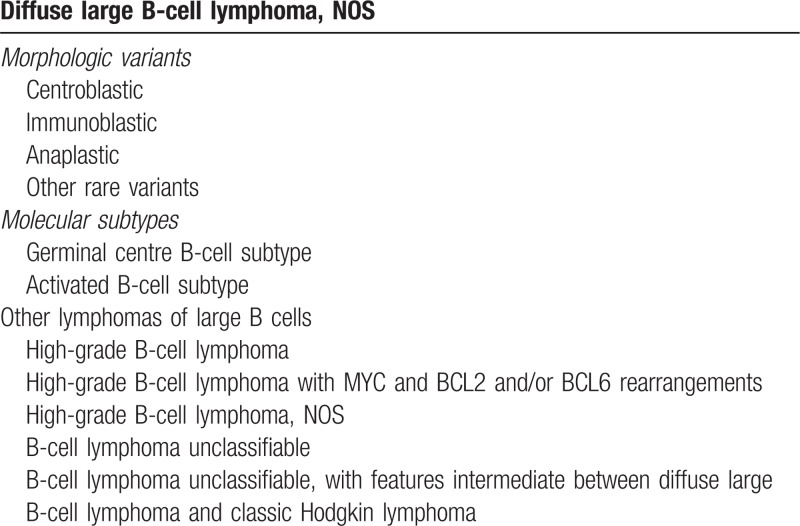
DLBCL Classification According to WHO^1^

## Cell of origin in DLBCL NOS

In year 2000, Alizadeh et al reported that DLBCL patients analyzed using gene expression profiling (GEP) could be divided in subgroups according to the cell of origin (COO).^2^ They identified three subgroups according to diversity in gene expression: the germinal Centre B-cell-like (GCB) in 40% to 50% of patients, the Activated B-Cell-like (ABC) in 50% to 60% of patients and an unclassifiable group. The distinct subgroups present gene expression patterns indicative of different stages of B-cell differentiation, and patients with GCB lymphoma has a better survival in comparison with ABC patients. Subsequent studies confirmed the different biological characteristics and clinical behavior according to the COO classification.^3,4^ The ABC subtype is driven by frequent mutations in the B cell receptor and NFKB pathways, as well as in CARD11, CD79a/CD79b and MYD88. MYD88 L265P mutation is present in 29% of ABC patients and is associated with extranodal disease (breast, testis, stomach, central nervous system) and poor outcome.^5^ In contrast, the GCB subtype is characterized by frequent mutations in phosphoinositide 3-kinase (PI3K) and apoptotic pathways.

Since the GEP methods are not applicable in the routine practice, numerous immune histochemical (IHC) algorithms have been developed as surrogates of GEP to assign the COO. The Hans algorithm using IHC for CD10, BCL6 and MUM-1/IRF4 with a cut off of 30% of reactivity is the most used and divides DLBCL into GCB and non-GCB. The concordance of the Hans algorithm with GEP is approximately 76%,^6^ which limits its use as a tool for clinical trial or personalized treatment approaches. More recently, a new GEP technique was applied: the NanoString Research Use Only Lymphoma Subtyping (NanoStringTechnologies, Inc., Seattle, WA) based on the analysis of 20 genes on formalin-fixed paraffin embedded samples. The Lymph2Cx assay was tested and reported a concordance of >95% with standard GEP, with an advantage in term of turnaround time, feasibility (fresh biopsies not required) and costs.^7^ This new technology was used for the analysis of large cohorts of patients treated with R-CHOP regimen in large prospective trials, but has yielded discordant results. The British Columbia Cancer Agency showed better prognosis for patients with GCB profile^8^ whereas the German High-grade Non Hodgkin Study group analyzed samples of two large randomized trials (RICOVER-60 and R-MegaCHOEP) and failed to identify any difference between GCB and ABC subtypes.^9^ These controversial results should not be clearly justified, but they may be due to a selection of low-risk patients in the subgroup of patients evaluated in the German studies; it should be underlined that in the more intensive schemes, R-CHOP14 or R-Mega-CHOEP, the role of COO seems to be less important that in the standard R-CHOP21 series. More recently, the GOYA study analyzed prospectively survivals of 1418 patients treated with R-CHOP or Obinutuzumab-CHOP by COO subtype. The assignment to COO classes (GCB, ABC and unclassified) was performed by Nanostring and showed a significantly higher 3-year progression-free survival (PFS) for GCB subtype (75% vs 59% and 63% in other subgroups).^10^ The revised version of the WHO classification in 2016 integrated the COO in the B lymphoma classification and recommends the assessment of cell of origin in all cases of DLBCL at least according to the Hans algorithm, despite the caveat above. While several agents (bortezomib, ibrutinib, lenalidomide) have shown increased activity in ABC-subtype DLBCL, their incorporation in frontline therapy has not yet shown superiority.^11–14^ This suggests that the COO may not capture enough of the molecular heterogeneity of DLBCL to allow targeted therapy.

## Double hit and triple hit lymphomas

Double and triple hit lymphomas are new entities in the 2016 WHO classification and are included in the High Grade B-cell Lymphoma category. These tumors harbor a rearrangement of the MYC gene and of the BCL2 and/or BCL6 gene. Double hit lymphomas (DHL) comprise approximately 10% of all DLBCL, with MYC/BCL2 being the more frequent subtype (65% of DHL cases), followed by MYC/BCL2/BCL6 triple hit (THL) lymphomas (20% of cases) and by MYC/BCL6 DHL (10% of cases).^15–17^ Most DHLs are of the GCB subtype. Double and triple hit lymphomas are very aggressive, present with advanced stage disease frequently involving the bone marrow and extranodal sites such as central nervous system and have a very poor prognosis. Treatment of these patients with standard chemotherapy R-CHOP is associated with dismal outcome with a median overall survival of 12 months from diagnosis. Many attempts to ameliorate the prognosis of these patients have been investigated. While no molecularly targeted approach has yet shown success, several studies have suggested that intensified chemotherapy regimens can improve the outcomes of these patients.^18,19^ The very poor prognosis of patients with relapsed/refractory DHL/THL makes imperative the development of effective (and ideally molecularly targeted) frontline regimens. A better understanding of the fundamental biology and therapeutic vulnerabilities of these tumors is a major unmet clinical need at present.

## Double expressor lymphoma

MYC and BCL2 represent two oncogenes implicated in proliferation and apoptosis and are key regulators of DLBCL pathogenesis. As noted above, concurrent chromosomal translocations are associated with aggressive and refractory clinical behavior in DLBCL. However, other mechanisms can result in overexpression of the proteins on the tumor cell surface, independently of translocation events. The concomitant double expression of MYC and BCL2 is present in 20% to 30% of DLBCL; the group of DLBCL with these characteristics is termed double expressor lymphomas (DEL), but it is not a different biological entity. This phenotype is more frequent in the ABC subgroup. The cut off for positivity is still matter of debate, however, expression of BCL2 more or equal to 50% and MYC expression more or equal to 40% as originally reported is considered adequate.^1^ DELs have been associated with an inferior prognosis under RCHOP treatment.^15,20–22^ However, and despite the recognition of the adverse associated prognosis, the biology of DELs is still unclear; indeed, in the updated WHO this entity has not been assigned to a specific category and is grouped within DLBCL NOS. To date no specific treatment has been shown to mitigate this adverse prognosis. Of note, the single expression of MYC is present in 25% to 30% of lymphoma, irrespective of COO, and does not represent an adverse prognostic factor in patients treated with R-CHOP.^23^ In contrast, BCL2 overexpression is highly correlated with ABC subgroup, and in some series has been associated with inferior PFS, independently from international prognostic index (IPI) and COO.^24^

## Mutations and genetic subtypes

In DLBCL, gene expression profiling was first used to identify subgroups according to COO. In 2018 Schmitz et al^25^ published a multiplatform genomic analysis of 574 fresh frozen samples of DLBCL and identified 4 genetic subtypes within the GCB and ABC groups; because the genetic composition of unclassified DLBCL is unknown, they were enriched for mutations. The first subtype is characterized by MYD88 and CD79B mutations (MCD) and is more frequent among ABC lymphomas; the second subtype is characterized by BCL6 fusions and NOTCH2 mutations (BN2) and is represented in similar proportions among ABC and GCB; the third is characterized by NOTCH1 mutations (N1), and is present more frequently among ABC the last subtype is characterized by EZH2 mutations and BCL2 translocations (EZB), and is present more frequently in GCB. Overall, authors estimated that, on the basis of the gene-expression predictor classifications of MCD, BN2, N1, and EZB cases, these genetic subtypes would comprise 46.6% of cases. Furthermore, an analysis of clinical outcome was performed on among 117 patients treated with R-CHOP whose tumor was classified into one of the genetic subtypes. The 5-year overall survival (OS) differed significantly between the groups and was 26%, 36%, 65%, and 68% for MCD, N1, BN2, and EZB respectively.

In another study, Chapuy et al^26^ performed whole exome sequencing of 304 primary DLBCL to detect low frequency mutations, somatic copy number alterations and structural variants. They identified five distinct DLBCL subsets: cluster 5 lymphomas had frequent gains in chromosome 18q with increased expression of BCL2 and MALT1, as well as mutations in *CD79* and *MYD88; those tumors are often in the ABC group*; cluster 1 exhibited BCL6 single variants and mutations of NOTCH2 signaling and are also most often of the ABC type; cluster 3 is a poor risk GCB group with BCL2 translocations and alterations of PTEN; cluster 4 is a favorable risk GCB group with alterations in BCR/PI3K, JAK/STAT, and BRAF pathway; finally, cluster 2 is a COO-independent group with frequent biallelic inactivation of TP53, loss of CDKN2A and genomic instability.

Interestingly, the genetic subgroups identified independently by the 2 aforementioned studies are overlapping, with the cluster 5 corresponding to MCD, cluster 1 to BN2 and cluster 3 to EZB subgroups. These new molecular classifications likely offer more molecular homogeneity than the original COO classification, and indeed allow the splitting of GCB and ABC groups into biologically distinct subgroups. This should help in targeting specific vulnerabilities and allowing a more personalized treatment approach in patients with DLBCL. Such studies are now beginning, but ultimately the value of these classifications will need to be demonstrated in prospective trials.

## First-line treatment of advanced stage disease

The standard treatment of advanced stage DLBCL is the monoclonal antibody anti-CD20 rituximab in combination to cyclophosphamide-doxorubicin-vincristine-prednisone (R-CHOP).^27^ Despite the high rate of complete response (76%) at the end of the treatment and an impressive long-term outcome, with 10-year progression-free survival (PFS) of 37% and overall survival (OS) of 44%, roughly the 40% of the patients experienced relapse or progression.^28^

In young patients with advanced DLBCL at good prognosis (no risk factors or one risk factor according to age-adjusted International Prognostic Index, IPI, at stage II-IV disease, or stage I disease with bulk), the standard treatment was represented by six courses of R-CHOP.^29^ The updated MInT data, at a 6-years follow-up, suggested a central role of radiotherapy on bulky disease in less-favorable group of young patients.^30^ To investigate a potential survival benefit of a dose-intensive regimen compared to chemoimmunotherapy in young patients at low-intermediate risk (age-adjusted IPI 1), the French group conducted a randomized trial comparing dose-intensive rituximab, doxorubicin, cyclophosphamide, vindesine, bleomycin, and prednisone (R-ACVBP) versus standard R-CHOP. This study suggested that more aggressive treatment may improve outcome in young DLBCL patients at low-intermediate IPI risk, with raised but manageable toxicities.^31^ Indeed, in this study R-CHOP21 regimen was given without radiotherapy and the results obtained with R-ACVBP were superimposable to those obtained in the MInT trial with R-CHOP plus radiotherapy.

In order to ameliorate the prognosis of advanced DLBCL at high risk, 2 large randomized trials were conducted, to compare the standard R-CHOP every 21 days with a dose-dense R-CHOP performed every 14 days; the results were superimposable into the 2 arms.^32,33^ In young patients with DLBCL at poor prognosis according to international prognostic index, several options have been investigated to improve outcomes, including the introduction of consolidation with high-dose chemotherapy plus autologous stem-cell transplantation (ASCT) as part of the first-line treatment. Four randomized trials were conducted, comparing a full course of R-CHOP (or intensified R-CHOP) with or without intensification with high-dose chemotherapy followed by ASCT; the intensification was able to reduce failures in 2 studies, but no advantage in overall survival was shown. In the SWOG-9704 trial, 397 patients were treated with 5 courses of CHOP21 or R-CHOP21, and 253 responsive patients were randomized to receive 3 more cycles of CHOP21 or 1 cycle of CHOP21 followed by ASCT conditioned by total body irradiation or carmustine containing regimen. The study showed an improvement in PFS in transplantation group compared with no transplantation, but no benefit in OS was observed.^34^ A benefit in terms of Failure Free Survival (FFS) was reported by the phase 3 randomized trial FIL-DLCL04; in the Italian trial, 399 young patients with DLBCL at high-risk were randomized to receive a full course of chemo-immunotherapy at 2 different level of intensification (R-CHOP14/R-MegaCHOP14) or a short course of the same scheme followed by intensification with high-dose cytarabine, mitoxantrone and dexamethasone, followed by ASCT conditioned by BEAM (carmustine, cytarabine, etoposide, melphalan) regimen. An advantage in FFS at 2-year of 71% (95%, CI 64–77) in the transplantation group vs 62% (95%, CI 55–68) in the non-transplant group, hazard ratio (HR) 0.65 (95%, CI 0.47–0.91) was observed, but this difference did not translate in an advantage of OS.^35^ No differences in OS nor in PFS between transplant and no transplant were reported in other 2 studies: the German DSHNHL 2002-1 trial, comparing R-CHOP14 plus etoposide (R-CHOEP14) and the more toxic intensified R-MegaCHOEP14 followed by ASCT, and the Italian GITIL trial, comparing 8 cycles of R-CHOP14 with an high-dose sequential chemotherapy program.^36,37^

An alternative and intensive infusional regimen is the dose-adjusted EPOCH-R (etoposide, prednisone, vincristine, cyclophosphamide, doxorubicin, and rituximab), tested on untreated DLBCL in a phase II trial by The Cancer and Leukemia Group B (CALGB), obtaining a 4-year PFS of 81%.^38^ To demonstrate the superiority of DA-EPOCH-R compared to R-CHOP, the CALGB/Alliance conducted a phase III randomized study in 524 DLBCL patients. Authors concluded that, despite increase toxicity and complexity in DA-EPOCH-R treated patients, there was no improvement in PFS, OS, or response rate compared to standard R-CHOP. In a post hoc comparison of PFS by arm in subgroups of age, lactate dehydrogenase level, Eastern Cooperative Oncology Group performance status, extranodal disease, stage and IPI risk group, patients with intermediate-high IPI risk treated with DA-EPOCH-R seemed to have a benefit in term of PFS compared to those treated with R-CHOP, but it was an unplanned and not powered analysis.^39^

An unmet clinical need in the treatment of DLBCL was represented by the risk of relapse in the central nervous system (CNS), an uncommon but devastating event, that occurred in up to 10% of advanced DLBCL at high-risk. The addition of rituximab to standard chemotherapy has contributed to a reduction in both systemic and CNS relapse, but it was not enough.^40,41^ The best strategy to prevent CNS dissemination was not yet established; intrathecal injections of methotrexate seems to be a suboptimal method, whereas intravenous high-dose methotrexate has been shown to be associated with efficient disease control.^42^ Some data reported a reduced incidence in CNS recurrence in combined chemoimmunotherapy with biological drugs (lenalidomide, ibrutinib).^43,44^

## First-line treatment of double expressors, double or triple hit lymphomas

The outcome of DEL, DHL, and THL is unsatisfactory with standard R-CHOP. Nowadays, the single MYC rearrangement or the overexpression of Bcl2 and MYC, are not yet considered validated risk factors, and R-CHOP still remain the standard therapeutic option. Nevertheless, several studies reported the advantage of intensified treatment in this setting. DA-EPOCH-R was tested in 53 patients at high risk and with single hit (MYC) or double hit, obtaining durable remission, with 4-year EFS of 71% (95% CI 56,5–81,4) and 4-year OS of 76,7% (95% CI 62,6–86,1) for all patients.^45^

In a recent Italian study, 114 consecutive patients with double expressors DLBCL were identified and treated with DA-EPOCH-R in 51 and R-CHOP in 63 patients; in this series of patients, the treatment with DA-EPOCH-R was associated with a significant improvement of PFS and OS in young patients and an advantage in term of PFS in favor of DA-EPOCH-R was seen in all patients with genetic abnormalities (single translocations, atypical double hit and double or triple hit). The data are encouraging, but retrospective and thus need to validated in a prospective trial.^46^

Due to the rarity of the disease, there are no prospective trials in the setting of DHL or THL that represents a critical unmet clinical need. Due to the high proliferation and the risk of central nervous system (CNS) dissemination, R-CHOP is not adequate, with a median PFS of 7.3 months, and more intensive treatments, such as schemes used in Burkitt lymphomas containing high dose methotrexate and cytarabine are frequently used. Petrich et al^19^ published a pooled retrospective analysis in which patients with DHL were treated with R-CHOP or intensive induction therapy, which included the dose-adjusted R-EPOCH regimen, HyperCVAD/MA fractionated cyclophosphamide, vincristine, doxorubicin, dexamethasone, alternating with methotrexate and cytarabine, or CODOX-M/IVAC. Response rates in this non-randomized retrospective study were highest for dose-adjusted R-EPOCH. Intensive induction was associated with improved progression-free survival and improved overall survival on multivariable analysis.

The so called “Magrath scheme”, with alternate R-CODOX-M/R-IVAC (rituximab-cyclophosphamide, vincristine, doxorubicin, methotrexate, ifosfamide, etoposide, cytarabine) is an aggressive regimen developed for Burkitt lymphoma used in aggressive lymphomas. Twenty-five patients affected by DHL were treated at the British Columbia Cancer Agency (BCCA) with R-Magrath and achieved a CR rate of 36%, with a 2-year PFS of 47% (95% CI, 30- 64%) and a 2-year OS of 61% (95% CI, 46–76%).^47^

The MD Anderson Cancer Center reported a retrospective series of 129 patients; CNS involvement occurred in 13% of patients. The 2-year EFS in patients who received R-CHOP, R-EPOCH, and R-HyperCVAD/MA were 25%, 67%, and 32%, respectively.^18^

In patients obtaining a complete response after induction, a consolidation with high-dose chemotherapy and ASCT, not seems to determine a better PFS and OS, but no prospective or randomized data are available.^48^ Considering that the majority of DHL patients have more than 65 years at diagnosis and thus are not eligible for intensified treatments, DA-EPOCH-R scheme should be a feasible therapeutic option in the elderly.

## Salvage treatment in patients eligible to high dose treatment

Since the PARMA trial, in the pre-rituximab era, a high-dose cisplatin and cytarabine-containing regimen (DHAP) followed by high-dose chemotherapy and autologous stem cell transplantation was considered the standard treatment in relapsed/refractory DLBCL patients, with a 5-years OS of 53%.^49^ In patients eligible to intensive treatment, in relapsed and refractory setting, the high dose chemotherapy followed by ASCT is still the standard of care in the rituximab-era.^42^ The addition of rituximab to second-line chemotherapy followed by ASCT significantly improved PFS in patients not exposed to rituximab as part of their first-line treatment. In the CORAL trial, 396 patients with DLBCL relapsed/refractory after CHOP with/without rituximab, were randomized to receive rituximab, ifosfamide, carboplatin, and etoposide (R-ICE) or rituximab, dexamethasone, high-dose cytarabine, and cisplatin (R-DHAP); responsive patients were given BEAM plus ASCT.^50^ In the CORAL trial, 48% of the patients did not underwent transplantation, the majority due to lack of response to induction; this finding represent the most important limitation of the study. However, this study was helpful to identify prognostic parameters at relapse correlated to lower 3-years PFS and OS (*P* < .001): prior rituximab treatment, early relapse (within 12 months) and a high score age-adjusted IPI score. In particular, early relapse and prior rituximab exposure, identified a group at dismal prognosis, with 3-year PFS of 23%. However, even in this unfavorable population, patients who underwent ASCT showed a better outcome compared to those not underwent ASCT (3-year PFS of 39% vs 14%, *P* < 0.001). Among the 396 patients included into the trial, 249 patients had histologic material available, and were studied for COO according to Hans profile. Based on COO assessed by immunohistochemistry, patients with non-GCB have a worse prognosis compared to GCB irrespective to R-DHAP or R-ICE chemotherapy; furthermore, GCB-like patients have an improved outcome when treated with R-DHAP compared with R-ICE in the CORAL randomized trial.^51^

In high-grade B-cell lymphoma, patients that experienced relapse and refractory disease after front line treatment had a dismal prognosis irrespective to the second line approach, and no standard therapeutic approach has been defined.^52^

Herrera et al, retrospectively studied the impact of DEL or DH status in a multicenter cohort of 78 patients (40% DLBCL, 47% DEL, 13% DHL) relapsed or refractory, who underwent allogeneic stem cell transplantation (alloSCT). There were no significant differences in 4-year PFS or OS between patients with DEL compared with patients without DEL (PFS 30% versus 39%, *P* = 0.24; OS 31% versus 49%, *P* = 0.17) or between patients with DHL compared with those without DHL (PFS 40% vs 34%, *P* = 0.62; OS 50% vs 38%, *P* = 0.46). In this series of patients, alloSCT was able to produce durable remissions in patients with relapsed or refractory aggressive B-NHL, irrespective of DEL and DHL status.^53^

In patients not eligible to high dose chemotherapy plus transplantation or in patients with refractory disease or relapsed after autologous transplant, the prognosis is very poor with standard chemotherapy and novel agents should be considered.^54–57^

## Novel salvage treatments

### Targeted therapy

While current prognostic biomarkers, including COO classification, have not clearly demonstrated which patients will respond to targeted therapies, novel genomic classifications have the potential to improve incorporation of these drugs into personalized treatment strategies.

B-cell receptor inhibitors. Bruton tyrosine kinase (BTK) is a member of the TEC family of kinases, which has been identified as a key signaling component downstream of the B-cell receptor (BCR).^58^ Abnormalities in *BTK* were originally found to drive the pathogenesis of X-linked agammaglobulinemia (XLA), an immunodeficiency associated with inhibition of B-cell development, and later linked to the B-cell malignancies.^59^ This finding led to the development of the first-in-class, oral, covalent BTK inhibitor, ibrutinib, as well as next generation BTK inhibitors, such as acalabrutinib. It has been hypothesized that ABC subtype DLBCLs have increased sensitivity to BTK inhibition given dependence on constitutive NF-кB signaling, a downstream effector of BTK, for survival and proliferation.^60^ Early phase trials supported preclinical data, with the phase I/II trial of ibrutinib monotherapy in relapsed/refractory DLBLC resulting in an ORR of 37% (14/38) in patients with ABC DLBCL, but only 5% (1/20) in patients with GCB DLBCL (*P* = 0.0106).^61^ Specifically, among patients with ABC subtype DLBCL, responses were seen in 3/5 patients with *CD79B* mutations, 4/4 patients with *CD79B* and *MYD88* mutations, 0/4 patients with isolated *MYD88* mutations, and 0/3 patients with *CARD11* mutations.^61,62^ The median progression-free survival (PFS) in responding patients was 5.5 months. Despite this encouraging early-phase data, the randomized trial Phoenix in the frontline setting have failed to demonstrate a benefit of adding ibrutinib to chemotherapy in patients with non-GCB DLBCL.^13^ Several causes can explain this result: first of all, the COO profile was assessed by immunohistochemistry based on Hans’ algorithm and not by gene expression profiling or nanostring technologies; second, R-CHOP plus placebo treated patients has an impressive outcome, making the hypothesis of a bias selection in enrolled patients into the trial; last but not least, elderly experienced more toxicities compared to young patients, determining premature discontinuation of the treatment. The exploratory analysis conducted on patients younger than 60 years, showed a benefit of adding ibrutinib to R-CHOP in term of EFS, PFS, and OS, compared to R-CHOP alone. The risk profile for ibrutinib plus R-CHOP seems to be age dependent, maybe due to multiple factors, including immune suppressive effects of iBTK. Further studies are needed to confirm this data. A deeper understanding of the genomic underpinnings of DLCBL, however, suggest a potential role of BTK inhibitors in the correct genomic context. Patient with *CD79B MYD88*^L265P^double mutations (MCD) or those with near-uniform *BCL2* copy gain, frequent activating *MYD88*^L265P^ and *CD79B* mutations (cluster 5), for example, may benefit from the incorporation of targeted BTK inhibition in their lymphoma treatment.^25,26^ Additional studies aimed at utilization of genomic signatures to guide the use of targeted therapy are required to confirm this possibility.

Additional downstream signaling components of B-cell receptor signaling include SYK and PYC. While again there was promising preclinical rationale for the role of SYC and PYC inhibitors, clinical trials in patients with DLBCL were disappointing. For example, a multicenter phase II study of fostamatinib, in which 68 patients with DLBCL were enrolled (58% GCB, 30% ABC, 12% unclassifiable), resulted in an ORR of only 3%.^63^ Similarly, inhibition of PKC-β, a downstream effector of multiple signaling pathways, had limited efficacy as both a salvage and maintenance therapy.^64^

Lenalidomide. Lenalidomide is an oral immunomodulatory drug with direct antineoplastic activity and immunologic effects. Preclinical data suggests that lenalidomide's anti-tumor properties include inhibition of tumor necrosis factor-α, vascular endothelial growth factor, and NFκB activity.^65^ In DLBCL, it has further been shown that anti-tumor effects were associated with downregulation of interferon regulatory factor 4 (IRF4), a hallmark of ABC DLBCL cells.^66^ These findings led to studies in aggressive non-Hodgkin lymphomas (NHLs), including patients with DLBCL.^67,68^ For example, an international, phase II trial of lenalidomide in 217 patients with relapsed or refractory aggressive NHL, led to an ORR of 35%.^68^ In the DLBCL cohort, the ORR was 28% with 7% complete response (CR) rate. Disappointingly, patients with DLBCL had the shortest median PFS and response duration of 2.7 and 4.6 months, respectively. Given limited activity of lenalidomide as a single agent, it has also been studied in combination with rituximab in patients with relapsed/refractory DLBCL, transformed follicular lymphoma and grade 3 follicular lymphoma.^69^ In this study, responses were slightly improved, with an ORR of 33% and median response duration of 10.2 months. As with other targeted therapies, lenalidomide has also been studied in combination with frontline chemotherapy R-CHOP. Phase II studies suggested that lenalidomide may mitigate the negative prognosis associated with non-GCB DLBCLs and true ABC DLBCLs assessed y GEP.^70–72^ Unfortunately, preliminary data of the Robust phase III randomized trial, investigating R-CHOP plus lenalidomide/placebo in ABC-DLBCL patients, showed no improvement in PFS by adding lenalidomide in this setting of patients; full analysis is ongoing.^14^

Proteasome inhibitors. Proteasome inhibitors, such as bortezomib, have also been proposed to increase activity in ABC DLBCLs. Bortezomib acts by blocking degradation of IκBα, consequently inhibiting NF-κB activity. Initial studies have shown limited activity of bortezomib as a single agent in relapsed/refractory DLBCL. However, when bortezomib was combined with chemotherapy, a small study demonstrated increased responses (83% versus 13%) and median OS (10.8 months versus 3.4 months) in ABC as compared to GCB DLBCL.^73^ It should be noted that this study was small with only 12 patients with ABC and 15 patient CGB subtype DLBCL. The addition of bortezomib in combination with R-CHOP for initial therapy of DLBC also showed a benefit of proteasome inhibition in high-risk patients.^11^ Based on these encouraging preliminary studies, a randomized phase II trial comparing R-CHOP to R-CHOP plus bortezomib in patients with non-GCB subtype DLBCL was performed.^12^ The results of this study were disappointing (from a personalized therapy perspective) with an ORR of 98% and 96% and 2-year survival rate of 88.4% and 93.0%, with R-CHOP and R-CHOP plus bortezomib, respectively.

Anti-apoptotic inhibitors. Anti-apoptotic inhibitors are an exciting class of therapy with efficacy across a range of hematologic malignancies. Given frequent BCL-2 over-expression and the presence of *BCL-2* translocations in DLBCL and high-grade B-cell lymphomas, both correlated with unfavorable outcome, there has been a particular interest in studying the BCL-2 inhibitors, such as venetoclax, in these diseases. While venetoclax was found to be highly active in chronic lymphocytic leukemia (CLL), with rapid onset of tumor lysis in the phase I study, results were more modest in DLBCL.^74^ The phase I study of venetoclax in patients with NHL enrolled 109 patients, 34 of whom had DLBCL. In the DLBCL subset, there was an ORR of 18% with a CR rate of 12%, suggesting that while the majority of patients did not respond, there may be limited subset of patients with sensitivity to BCL-2 inhibition.^75^ While the COO, BCL-2 expression, or *BCL-2* translocation status of the patients on this trial were not known, efforts to identify biomarkers of response are ongoing. For example, the phase II CAVALLI study has studied the addition of venetoclax to R-CHOP in 208 patients with DLBCL and with a non-randomized comparison with the Goya study population, the study demonstrated that this association may be most beneficial in patients with BCL-2 overexpression or translocation, though randomized trials will be required to confirm these findings.^76^ Recent preclinical work has also identified genomic subtypes that may be most susceptible to BCL-2 inhibition. Specifically, in vitro and in vivo models representative of the recently described cluster 3, which consisted of high-risk GCB DLBCLs with *BCL2* SVs, inactivating mutations and/or copy loss of *PTEN* and alterations of epigenetic enzymes, as well as cluster 5, which consist of high-risk ABC DLBCLs with near-uniform *BCL2* copy gain, frequent activating *MYD88*^L265P^ and *CD79B* mutations, are more sensitive to BCL2 inhibition, especially in combination with PI3Kα/δ blockade.^77^

### Immunotherapy

In recent years, the advent of CAR-T cells has revolutionized the treatment of relapsed/refractory DLBCL. Bispecific antibodies, which provide a theoretically similar mechanism of anti-tumor activity, are also in active development.

CAR-T cells. Anti-CD19-directed chimeric antigen receptor (CAR) T-cells are acquiring a role in treatment of DLBCL relapsed after or refractory to chemotherapy. Since first use in humans in 2010,^78^ many efforts have been made to develop new generations of CAR-T-cells and to better understand possible clinical applications and toxicities. Today two different products, axicabtagene ciloleucel (axi-cel) and tisagenlecleucel (t-cel) have been approved from FDA and EMA for treatment of refractory or relapsed DLBCL patients after at least two prior lines of systemic therapy. A third product, the lisocabtagene maraleucel (liso-cel) is anticipated to be approved by FDA in 2019. Differently from other new compounds, this new treatment is not an “off-the-shelf” drug, indeed the availability of CAR-T-cells is not immediate. Infact the T-cells are collected from the patients, then the cell product is genetically modified and then is reinfused to the patients.

Axi-cel contains a CD28 costimulatory domain in addition to a CD3 zeta domain. The Phase 2 ZUMA-1 study enrolled 111 patients and 101 of them received axi-cel.^79^ Overall Response rate (ORR) was 82% and 54% of patients achieved a CR, among 77 DLBCL patients, CR was achieved in 49%. CR was durable at a median follow-up of 15.4 months in 70% of patients. The updated follow-up at 2 years for these patients showed that the median OS is not reached and 39% of patients have ongoing responses 2 year after the infusion.^80^ Recent data showed an ORR of 81% and CR of 57% at 90 days among 274 patients treated outside clinical studies with the axi-cel commercial product^81^ in 17 US centers. Real life toxicity profile was also similar to the ZUMA-1 study. T-cell contains a 4-1BB costimulatory domain. Following promising results observed in a Phase I study, a large multicenter phase II study was conducted, the JULIET trial.^82^ Among 106 infused patients the ORR was 50% and CR rate was 32%, in the subgroup of 16 high grade lymphoma patients the ORR was 50% indicating that the clinical response was independent from the genetic lesions. Liso-cel has a 4-1BB costimulatory domain and is engineered so that the final product has a defined composition of CD4 and CD8 T-cells. Among 73 evaluable patients, ORR was 80% and CR rate was 59%.^83^ Administrations of these products is correlated to a remarkable toxicity. Cytokine Release Syndrome (CRS) and Neurotoxicity (NT) are fearsome and potentially life-threatening. Comparison of toxicity incidence and grading between different products is not easy because of the different toxicity-scale used in the studies. However, all grade CRS incidence is ranging between 37% and 93% with a percentage of Grade≥3 in 1 to 22% of patients. The rate of NT of all grades is ranging between 23% and 65% with grade more than 3 in 12 to 31% of patients. Majority of toxicities resolved in a median of 15 days using immunosuppressive drugs like the anti-IL6 receptor antibody (Tocilizumab) and steroids in case of CRS and corticosteroids alone in case of neurotoxicity. Today, considering all patients affected by DLBCL treated with CAR T-cells the rate of mortality related to the treatment is estimated around 2% to 4%. Infections and prolonged cytopenias have been also reported.

Based on the data available to date, it appears that the anti-tumor immune activity of CD19-CAR-T cells is independent of molecular profile for DLBCL. Indeed, in the ZUMA-1 study, the ORR in patients with DEL and HGBL was 91%, with a CR rate of 70%, similar to that in the entire cohort.^80^ DHL/THL was not broken out as a subgroup in this analysis. However, in the JULIET trial, patients with DHL/THL comprised a significant proportion of the cohort (19/70, 27%), and their ORR was the same as patients without DHL/THL.^82^ In addition, the ORR of patients with GCB DLBCL was also very similar to that of patients with ABC DLBCL. Comparing to standard population of R/R DLBCL, patients included into these trials appear to be younger and with better performance status, suggesting a selected population, while additional experience is necessary to definitively answer this question, it seems that CD19-CAR-T is a powerful therapy for all forms of DLBCL, regardless of COO and more importantly regardless of DHL/DEL status. It is tempting to speculate that this remarkable feature of CAR-T treatment could apply more broadly to immunotherapy, making this potentially a cornerstone of future treatment of these high-risk DLBCL variants.

Bispecific antibodies. Bispecific T-cell engagers (BiTEs) antibodies consist of 2 single-chain variable fragments specific for CD3 and a tumor antigen; when the antibody binds to the antigen, cytotoxic T-cells are directed against the tumor cell resulting in cell lysis. Considering the characteristic expression of specific antigen such as CD19 and CD20 in B-cell malignancies, they are a natural target for the development of this type of immunotherapy. Differently from the adoptive T-cells immunotherapy with CAR T-cells, BiTEs are an off-the-shelf drug available without need of patient-dedicated manufacturing. The development of bispecific antibodies is exploring a broad range of tumor antigens as targets for new antibodies with potential applications in myeloma, leukemia and lymphoma patients.

Blinatumomab was the first-in-class CD19-specific BiTE approved by FDA in 2014 for the treatment of patients with relapsed or refractory acute lymphoblastic leukemia. Considering the expression of CD19 by lymphoproliferative diseases derived from the B lineage, efficacy of this drug in DLBCL patients has been tested. In 2016 a phase I study showed an ORR of 69% among 76 relapsed and refractory lymphoma patients treated with Blinatumumab at escalating dose. DLBCL patients were 14 with an ORR of 55% and a median duration of response of 13.5 months.^84^ A similar response rate of 43% with 19% of complete response was observed among 21 DLBCL patients treated in a Phase II study.^85^ Many others new bispecific antibodies directed against CD19 and CD20 are under evaluation in clinical trials involving patients with different lymphoma hystologies. Preliminary results show promising results also in patients with refractory and relapsed aggressive lymphomas with durable response in patients achieving complete remissions (Table 2).^86-88^ Common adverse events reported during treatment with bispecific antibodies are the cytokine release syndrome and the neurological toxicities. Many patients present fever and chills, more severe reactions with hypotension and multiorgan failure due to the macrophage activation are less frequent and are reported only during the first cycle. Neurological adverse events have been reported in 50% of patients with symptoms ranging from tremors to seizures or encephalopathy.

**Table 2:**
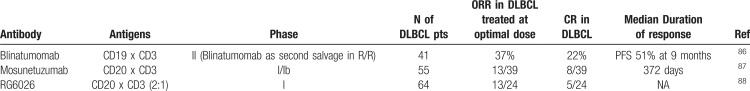
More Recent Abstracts on Bispecific Antibodies in DLBCL Treatment

To date, there is very little information on the response of specific DLBCL subgroups, in particular DHL/THL or DEL, to bispecific antibodies. The results of CAR-T cells lend hope that bispecifics could also be equally efficacious in those subgroups, but such speculation awaits confirmation from larger studies.

## Conclusions and future approaches

**Figure 1:**
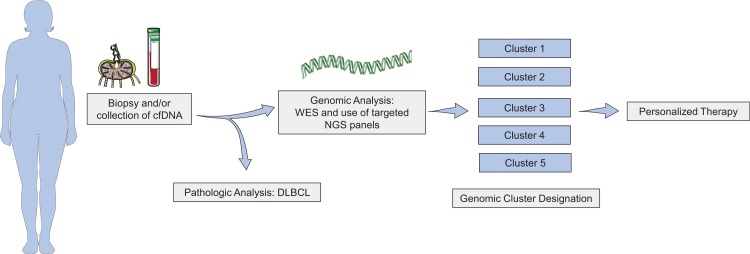
**Potential role of genomic profiling to guide precision medicine strategies in DLBCL**.

In recent years, there has been a dramatic expansion in our knowledge regarding the biology and heterogeneity of DLBCL, as well as an expansion of available therapeutic options, with new availability of both immune-based and targeted treatments. However, despite significant progress, particularly with the advent of CART cell therapy, there remains a subset of patients who are less likely to achieve durable response with frontline therapy, and still today only a minority of patients with relapsed or refractory disease will achieve a cure. While providing a useful starting point for categorization, the COO classification has to date not allowed the optimization of front-line or curative-intent salvage therapy, and no specific therapy has been developed for DHL/THL or DEL. Recent genomic analyses have shed more light on the genomic complexity of DLCB and promise a better possibility of personalized therapy (Fig. 1). In addition, such work may refine our biological understanding of MYC-deregulated DLBCL, which may betray an additional layer of biological complexity that interacts with the broader genomic profile. This could allow rational combinations to be tested in precisely defined subsets. It may be hoped that this body of work will fuel efforts focused on validating and refining genomic classification structures, biomarkers of response, and developing effective targeted therapies, that could be used in combination with chemotherapy, with each other, or with the powerful new tools of immunotherapy.

To this end, it will also be necessary to develop broadly available and affordable sequencing tools. Furthermore, given the aggressive nature of DLBCL, genomic information and classification must be available in a short time frame in order to be clinically relevant. Further improvements in sequencing platforms, the development of targeted sequencing panels, and use of cell free DNA (cfDNA), which could abrogate the need for tumor biopsies, have the potential to improve the process of genomic classification and utility of these technologies to improve care for patients with DLBCL.^89^

Ultimately, such broad and multifaceted efforts are likely required if we aim to cure all patients with DLBCL.
